# Co-Expression and Co-Purification of Archaeal and Eukaryal Box C/D RNPs

**DOI:** 10.1371/journal.pone.0103096

**Published:** 2014-07-31

**Authors:** Yu Peng, Ge Yu, Shaoxiong Tian, Hong Li

**Affiliations:** 1 Institute of Molecular Biophysics, Florida State University, Tallahassee, Florida, United States of America; 2 Department of Chemistry and Biochemistry, Florida State University, Tallahassee, Florida, United States of America; Max-Planck-Institute for Terrestrial Microbiology, Germany

## Abstract

Box C/D ribonucleoprotein particles (RNPs) are 2′-O-methylation enzymes required for maturation of ribosomal and small nuclear RNA. Previous biochemical and structural studies of the box C/D RNPs were limited by the unavailability of purified intact RNPs. We developed a bacterial co-expression strategy based on the combined use of a multi-gene expression system and a tRNA-scaffold construct that allowed the expression and purification of homogeneous archaeal and human box C/D RNPs. While the co-expressed and co-purified archaeal box C/D RNP was found to be fully active in a 2′-O-methylation assay, the intact human U14 box C/D RNP showed no detectable catalytic activity, consistent with the earlier findings that assembly of eukaryotic box C/D RNPs is nonspontaneous and requires additional protein factors. Our systems provide a means for further biochemical and structural characterization of box C/D RNPs and their assembly factors.

## Introduction

**Figure 1:**
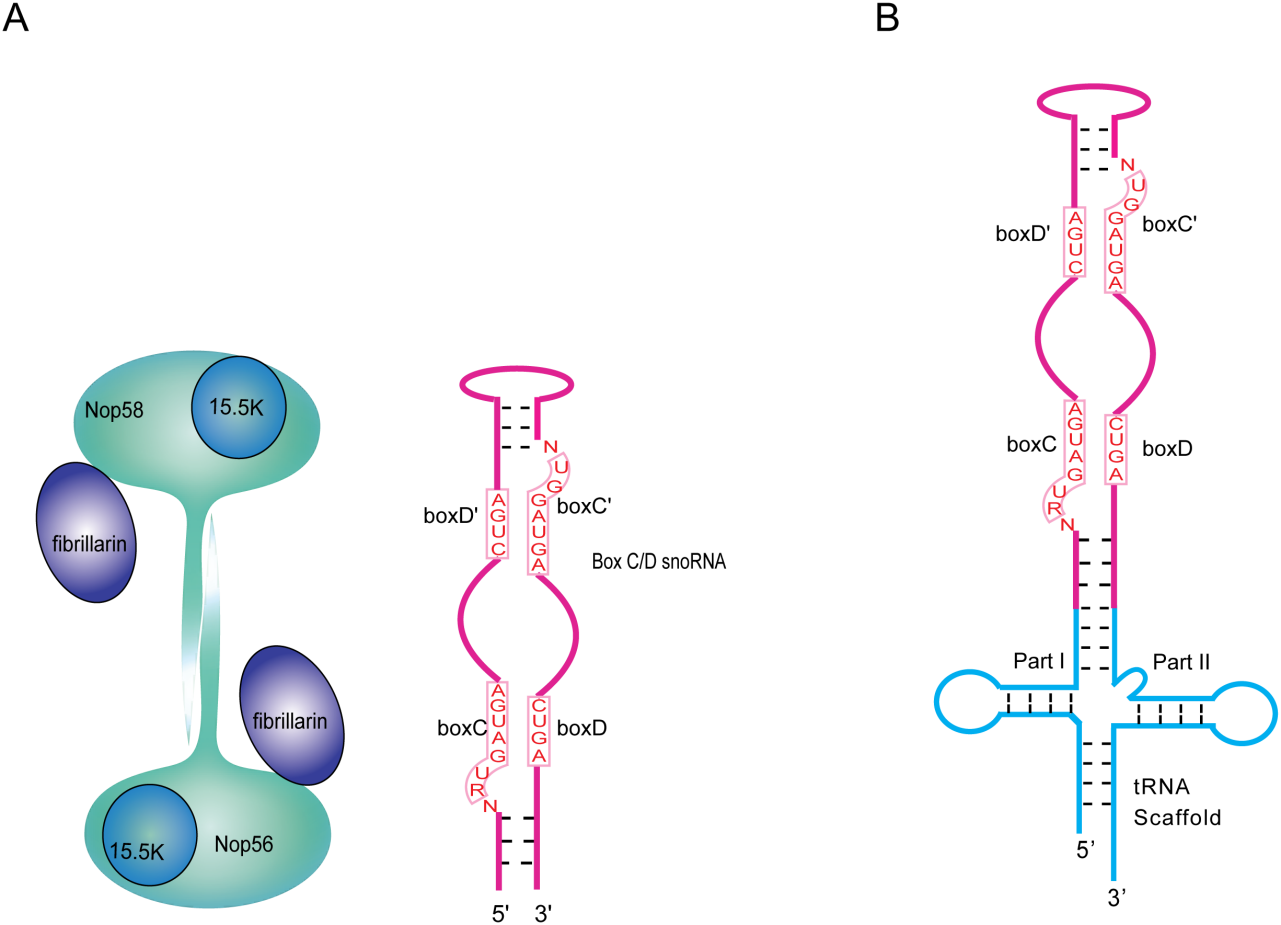
Schematics of box C/D ribonucleoprotein particles (RNPs) and chimera RNA used in co-expression studies. A) Eukaryotic box C/D RNPs comprise Nop56, Nop58, fibrillarin (FIB), 15.5 K (Snu13p in yeast), and a box C/D RNA. In archaea, Nop56 and Nop58 have a single homolog, Nop5, and 15.5 K has a homolog, L7Ae. The protein assembly model is based on architecture revealed by archaeal box C/D RNP crystal structures. B) Construction of the tRNA-box C/D RNA chimera. tRNA is divided into two parts (part I and part II) that flank the sequence encoding the box C/D RNA.

Box C/D small nucleolar ribonucleoprotein particles (snoRNPs) are one of the two major types of RNPs required for ribosome and spliceosome maturation. Large majorities of box C/D snoRNPs catalyze 2′-O-methylation of ribosome and spliceosome RNA by an RNA-guided mechanism [1], [2]. Other members of the box C/D snoRNPs participate in processing ribosomal RNA [3]–[5] or have microRNA-like functions [6]–[8]. A box C/D RNP comprises at least three (archaea) or four (eukarya) core proteins and a box C/D RNA. In archaea, the core proteins include fibrillarin, L7Ae and Nop5, while in eukaryotes they include fibrillarin, 15.5 K (homolog of L7Ae), Nop56 and Nop58 (homologs of Nop5) (Figure 1A). Fibrillarin is responsible for the methylation process by transferring the methyl group from a bound *S*-adenosyl-methionine (SAM) molecule to the 2′-hydroxyl group of the target RNA nucleotide. The Nop5 or Nop56/Nop58 proteins form a homo (archaea) or a hypothesized hetero (eukarya) dimer that is regarded as the scaffold of the complex [9]–[12]. L7Ae/15.5 K binds directly to the box C/D motif of the RNA, and in the case of the archaeal complex, it has been shown to initiate box C/D RNP assembly upon shaping the characteristic box C/D motif for binding by the Nop5 and fibrillarin heterodimer [11]–[17]. In general, eukaryotic proteins are more complicated than their archaeal counterparts and contain additional domains such as the GAR domain in fibrillarin and KKE/D repeats in Nop56/58 [18], [19]. Unlike their archaeal counterparts, eukaryotic snoRNPs are unable to assemble spontaneously. They require a complicated process mediated by assembly complexes to assemble [20]–[23].

Structural and biochemical studies of the box C/D RNPs depend on our ability to assemble and to purify the functional complexes. These processes have been more difficult with eukaryotic than with archaeal box C/D RNPs, owing to challenges in preparation of soluble eukaryotic proteins. Furthermore, heterogeneously assembled box C/D RNPs in cells hinder the purification of a single native box C/D RNP. As a result, current structural models of box C/D RNPs come from crystallographic and electron microscopic studies of archaeal box C/D RNPs reconstituted from individually purified components. These studies yielded two very different organizations of the particle. In the mono-RNP model, the L7Ae-bound box C/D RNA lies along the homodimer of the Nop5-fibrillarin complex in which fibrillarin is placed near each guide-substrate duplex [24]. In the di-RNP model, two box C/D RNAs, each bound with two L7Ae, lie across two parallel homodimers of the Nop5-fibrillarin complex [25], [26], leading to a square-shaped architecture. A recent combined study using small-angle x-ray scattering and nuclear magnetic resonance further supports the di-RNP model and reveals large conformational changes of the particle required for placing fibrillarin sequentially to each of the two target sites [27]. It is unclear whether the observed structural differences in archaeal box C/D RNPs are a result of different species used or sample preparation methods.

To facilitate studies of intact box C/D RNPs assembled under more native conditions, a bacterial expression system that permits co-expressing and purifying all proteins and the associated box C/D RNA is explored. This development is based on the co-expression strategies that greatly increase the stability and solubility of both proteins and RNA [9], [28]. In addition, the inclusion of a tRNA scaffold at either end of the RNA of interests further increases the stability of the RNA in *E. coli* [28]–[30]. Here, we show that both archaeal and human box C/D RNPs can be co-expressed and co-purified to homogeneity. However, a 2′-O-methylation assay showed that only co-purified archaeal box C/D RNP is catalytically active, suggesting a requirement for other conditions such as assembly factors or cellular environment for production of active human RNPs. We believe that the co-expression method can be used for studying archaeal box C/D RNPs under a more native condition as well as the assembly factors for human box C/D RNPs.

## Materials and Methods

### Plasmid construction

We used pQlink cloning system [31] (Addgene) as the platform for co-expressing box C/D proteins and RNA. To construct the archaeal box C/D RNP plasmid, pQafCDtRNA+, pQLink was inserted with the coding sequences of *Archaeoglobus fulgidus* (Af) Nop5, fibrillarin, L7Ae and an sR3 RNA. To construct the human box C/D RNP plasmid, pQhsCDtRNA+, pQLink was inserted with the coding sequences of *Homo sapiens* (Hs) NOP56 (amino acids 1 to 411), NOP58 (amino acids 1 to 401) and fibrillarin (amino acids 83 to 316), 15.5 K, and U14 snoRNA. Each protein-encoding gene was first cloned into pQLink-N plasmid using *BamH1* and *NotI* sites. The plasmids containing two different protein-encoding genes were then digested by *Swa I* and *Pac I*, respectively, treated with LIC qualified T4 polymerase (dCTP for *Pac I* digest and dGTP for *Swa I* digest) and then annealed at 70°C. Each clone was identified by digestion of the recombined plasmid with *Pac I.* This process was repeated for additional coding sequence until all were inserted.

**Figure 2:**
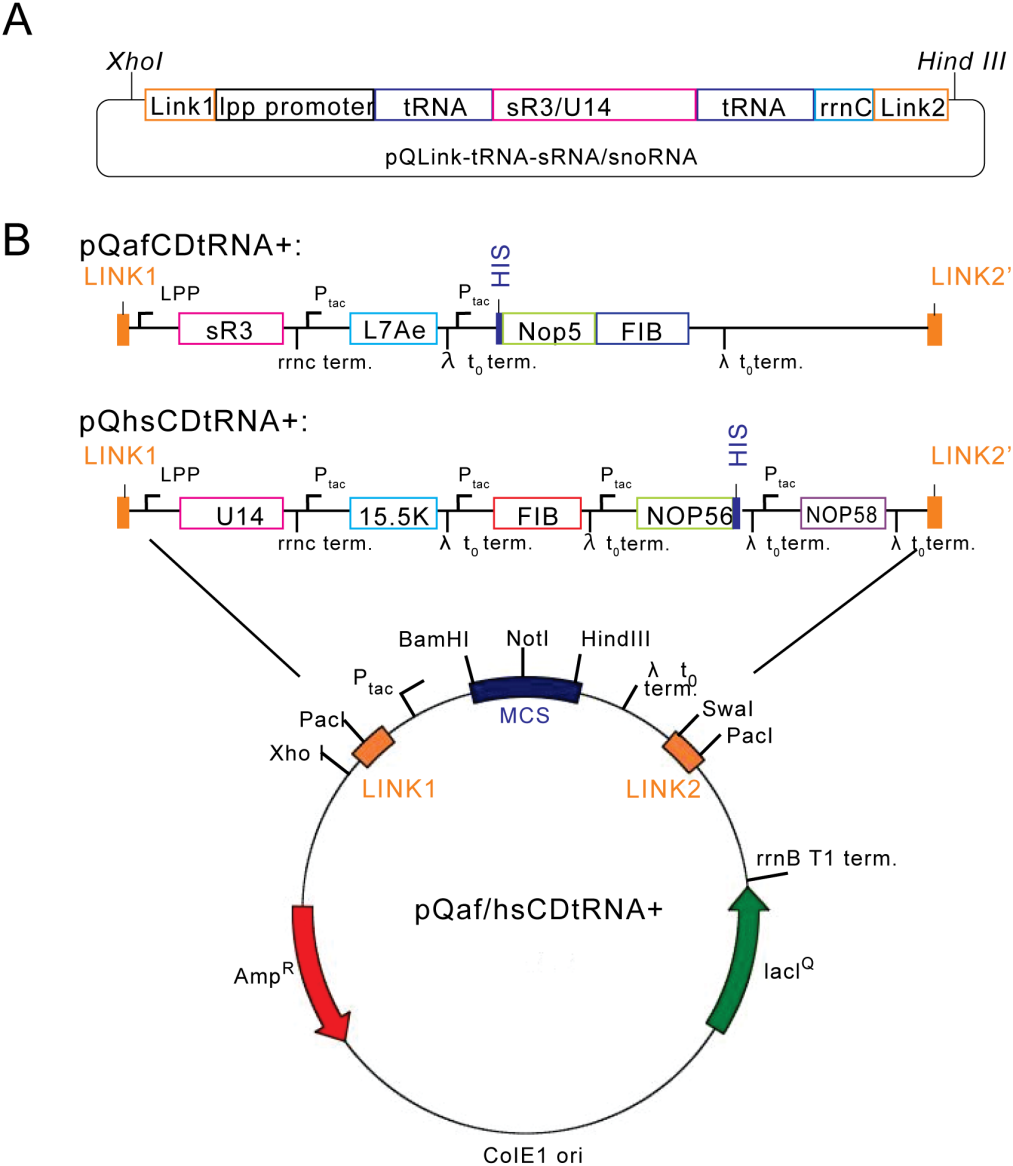
Diagrams of co-expression plasmids based on pQlink system. A). The pQlink-N vector inserted with tRNA-box C/D RNA chimera coding sequence. B). Co-expression plasmids inserted with all proteins and RNA encoding sequences. The region containing inserted genes is highlighted on top for pQafCDtRNA+ and pQhsCDtRNA+, respectively. Other elements of the co-expression plasmid are also shown at the bottom.

To construct tRNA-RNA chimera, DNA sequences of Af sR3 sRNA and human U14 snoRNA were fused with that of *E. coli* initiator tRNA^Met^ scaffold under the control of lpp promoter. The fused mini gene sequences contain the *Xho I* site, the LINK1 sequence (required for pQLink-N fusion), the lpp promoter, tRNA scaffold part I, sR3 sRNA/U14 snoRNA, tRNA scaffold part II, the rrnC terminator and the *Hind III* site (Figure 2A). The *Xho I* and *Hind III* sites were used to clone the mini gene into the pQLink-N vector. Finally, the tRNA-sRNA/snoRNA segment was recombined with box C/D protein coding sequences via the LINK reaction of the pQLink system resulting in pQafCDtRNA+ or pQhsCDtRNA+ plasmids (Figure 2B) [31]. We also cloned the box C/D RNA without the use of the tRNA scaffold, pQafCDtRNA− and pQhsCDtRNA−, for Af and Hs box C/D RNP, respectively. In addition, plasmids containing all protein-encoding sequences without an inserted RNA mini gene, pQafCD and pQhsCD, were also constructed.

### Purification of RNPs

The plasmids encoding the Af box C/D components were transformed into *E. coli* Rosetta (DE3) strain and those encoding the Hs box C/D components were transformed into the Nico21 (DE3) cells [32]. Both types of cells were grown to the optical density of 0.6–0.8 before being induced by addition of 0.2 mM Isopropyl β-D-1-thiogalactopyranoside (IPTG) and were grown for additional 18 hours at 16°C. Cells were harvested by centrifugation and stored in −80°C until purification. To purify the recombinant human U14 box C/D RNPs, cells were lysed with microfluidizer in a lysis buffer containing 25 mM Na_2_HPO_4_, pH 7.6, 500 mM NaCl, 10% glycerol, 0.5% Triton X-100, 2 mM phenylmethylsulfonyl fluoride (PMSF), 15 mM β-mercaptoethanol (β-ME). Cell debris was cleared by centrifuge at 21000 rpm for 45 minutes and supernatant was loaded on to a Ni-NTA column equilibrated with the lysis buffer. The column was washed extensively using the lysis buffer containing 50 mM imidazole and the bound RNPs were eluted using the lysis buffer containing 250 mM imidazole. The elutant was further purified by gel filtration chromatography using a Superdex 200 column equilibrated with a buffer containing 25 mM Na_2_HPO_4_, pH 7.6, 500 mM NaCl, 10% glycerol and 15 mM β-ME. To purify Af box C/D sR3 sRNP, cells were lysed by sonication in the same lysis buffer above without Triton X-100 followed by heating at 70°C for 30 minutes. The cell debris was cleared by centrifugation and the sRNP was purified similarly as U14 snoRNP.

### Quantitative Reverse Transcription Polymerase Chain Reaction (qRT-PCR) and TA-cloning

To test the expression of recombinant box C/D RNA, total RNA from RNP-expressing cells was extracted by phenol/chloroform extraction. A 200-µl aliquot of phenol saturated with 10 mM MgCl_2_, 10 mM Tris-HCl, and pH 7.4 was added to the cells suspended in 180 µl lysis buffer (10 mM MgCl_2_, 10 mM Tris-HCl, pH 7.4). After 20-minute gentle agitation at room temperature, the aqueous phase was separated by centrifugation. Residual phenol was then removed by addition of equal volume of chloroform. RNA in the aqueous phase was ethanol precipitated by addition of 0.1 volume of 4.0 M NaCl and 2 volumes of cold dehydration ethanol. To verify the presence of box C/D RNA in the purified RNP, the Ni-NTA-affinity purified protein complex was phenol/chloroform extracted and the RNA was ethanol precipitated as described above. Each extracted RNA was reverse transcribed using SuperScript VILO cDNA Synthesis kit (Invitrogen). Quantitative PCR was performed using an Applied Biosystems 7500 Fast Real-Time PCR System, with Invitrogen SYBR Green PCR Master Mix and primers at 0.5 µM each in a 20 µl reaction. The reverse-transcription amplified PCR products were cloned into the pCR2.1 vector using Invitrogen TA-cloning kit and verified by DNA sequencing.

### Methylation assays

Target RNAs containing [α-^32^P]-labeled cytidine at methylation sites were prepared by in vitro T7 transcription and were used for methylation assays. Transcribed RNAs were purified by phenol/chloroform extraction and ethanol precipitation. Methylation reactions were initiated by adding 10 nmol sRNP/snoRNP and ∼5000 cpm [α-^32^P]-labeled target RNAs to a 20 µl reaction mixture containing 20 mM HEPES (pH 7.0), 500 mM NaCl, 1.5 mM MgCl_2_, 5 mM DTT, 1 mM SAM, and 10% glycerol. After incubation at 70°C for Af sRNP or 4°C, 22°C, and 37°C for Hs U14 snoRNP for 2 hours, RNA in the reaction system were extracted by phenol/chloroform again. Reacted and extracted RNA sample was then treated with 2 units of nuclease P1 at 37°C overnight. The final product was resolved on a PEI-cellulose thin layer chromatography (TLC) plate in isobutyric acid: NH_4_OH: H_2_O (50:1:30, v/v). TLC plates were exposed at −80°C on to a phosphor screen (GE Healthcare) and results were analyzed on a Storm 860 system (Molecular Devices).

## Results

### Coexpression and purification of intact box C/D RNPs

Previously characterized *Archaeoglobus fulgidus* (Af) sR3 and human U14 RNA were used for co-expression with their respective box C/D proteins (Figure 1). sR3 resides within the intron of Af tRNA^Trp^ precursor and was shown to be responsible for methylation of tRNA^Trp^ [33]. In vitro, sR3 RNA is efficiently assembled with box C/D proteins into an active RNA-guided methyltranferase [13]. U14 RNA is a dual functional RNA required for both 18S rRNA methylation and processing [4]. For both RNAs, we also adopted a stabilization method based on chimeric construction [29] in which tRNA sequences flank that of sR3 or U14 RNA (Figure 1B).

We used pQlink-N cloning system [31] as a platform for co-expressing box C/D proteins and RNA (Figure 2). In this system, expressions of RNA and individual proteins are controlled by separate promoters and terminators. For RNA, the constitutive lpp promoter and a strong rrnC terminator ensure their effective transcription without induction. For proteins, the Ptac promoter allows controlled expression induced by IPTG. The single affinity tag incorporated into AfNop5 or HsNOP56 allows isolation of intact RNPs by affinity and size exclusion chromatography methods. For human fibrillarin, NOP56/58 and Af Nop5, we cloned full-length and several truncated fragments in order to obtain soluble complexes. Specifically, human fibrillarin without the GAR domain [34], NOP56/58 and Af Nop5 lacking the C-terminal charged tail (Figure S1) were found to be suitable for the co-expression and co-purification procedures.

**Figure 3:**
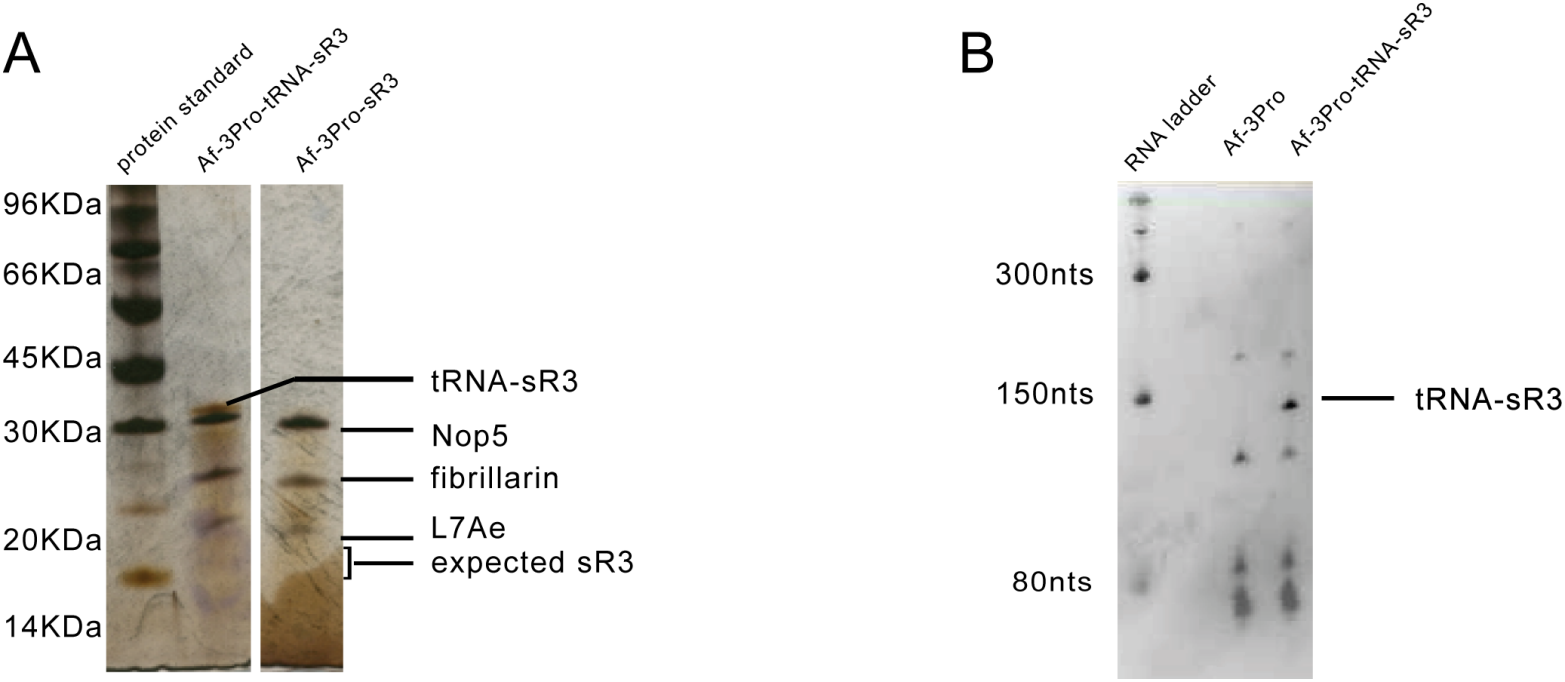
Co-expression and purification of recombinant *Archaeoglobus fulgidus* sR3 sRNP. A) Silver-stained SDS-PAGE gel analysis of purified sR3 sRNP components “Af-3Pro-sR3” denotes the sample expressing Nop5, fibrillarin, L7Ae and sR3 RNA. “Af-3Pro-tRNA-sR3” denotes that expressing Nop5, fibrillarin, L7Ae and sR3-tRNA chimeric RNA. Box C/D proteins and sR3-tRNA chimeric RNA are labeled and indicated by arrows. The sR3 sRNPs were purified by Ni-NTA affinity followed by gel filtration method based on the single histidine tag present in Nop5. B). Polyacrylamide gel analysis of total RNA extracted from cells expressing only box C/D proteins (Af-3Pro) or proteins plus sR3-tRNA chimeric RNA (Af-3Pro-tRNA-sR3). The location of sR3-tRNA chimera is indicated.

**Figure 4:**
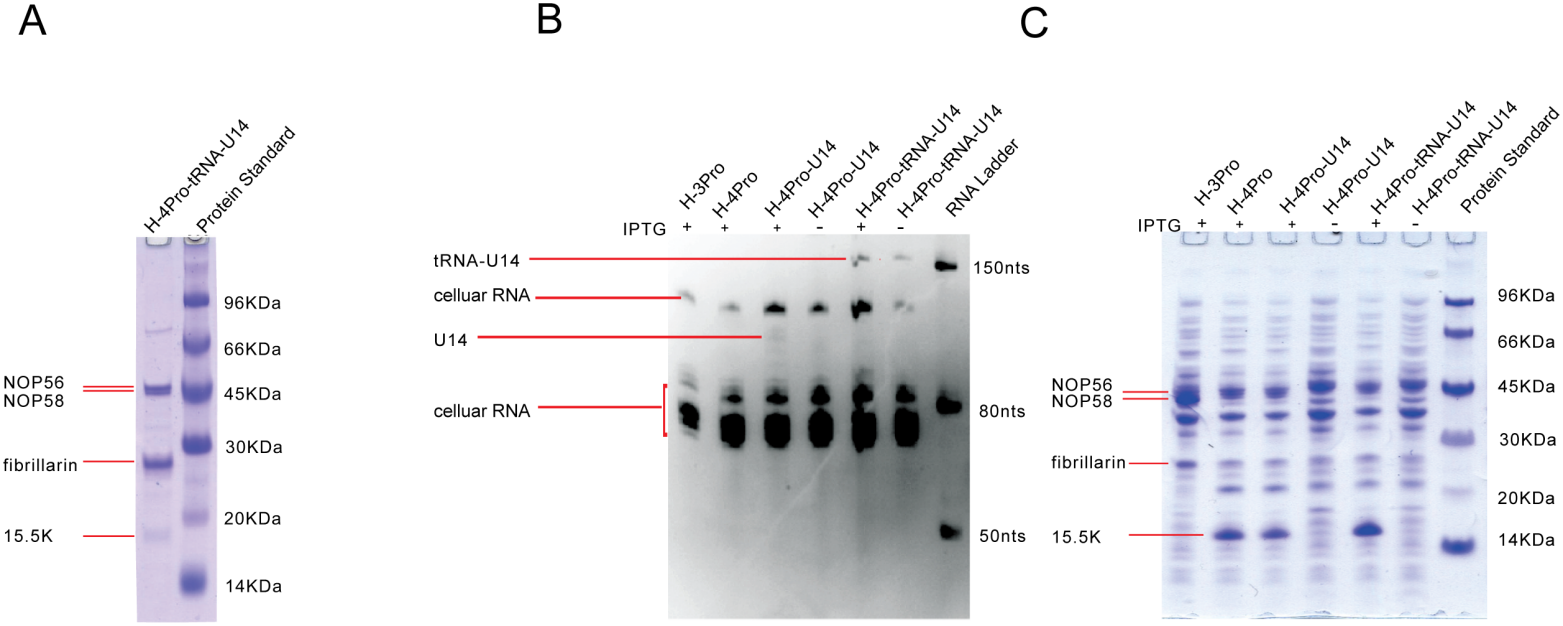
Co-expression and purification of recombinant human U14 box C/D snoRNP. A). Coomassie blue-stained SDS-PAGE gel analysis of purified U14 snoRNP. The U14 snoRNP was purified by Ni-NTA affinity followed by gel filtration method based on the single histidine tag present in NOP56. B). Total RNA extracted from cells expressing various proteins and RNA under both inducing and noninducing conditions. “H-3Pro” denotes the construct expressing NOP56, NOP58, and fibrillarin, “H-4Pro” denotes the construct expressing NOP56, NOP58, fibrillarin, and 15.5 K, “H-4Pro-U14” denotes the construct expressing NOP56, NOP58, fibrillarin, 15.5 K and U14 RNA, “H-4Pro-tRNA-U14” denotes the construct expressing NOP56, NOP58, fibrillarin, 15.5 K and U14-tRNA chimera RNA. C). Coomassie-stained SDS-PAGE gel analysis of the cell lysates of the samples described in B). Locations of NOP56, NOP58, fibrillarin and 15.5 K are indicated.

**Figure 5:**
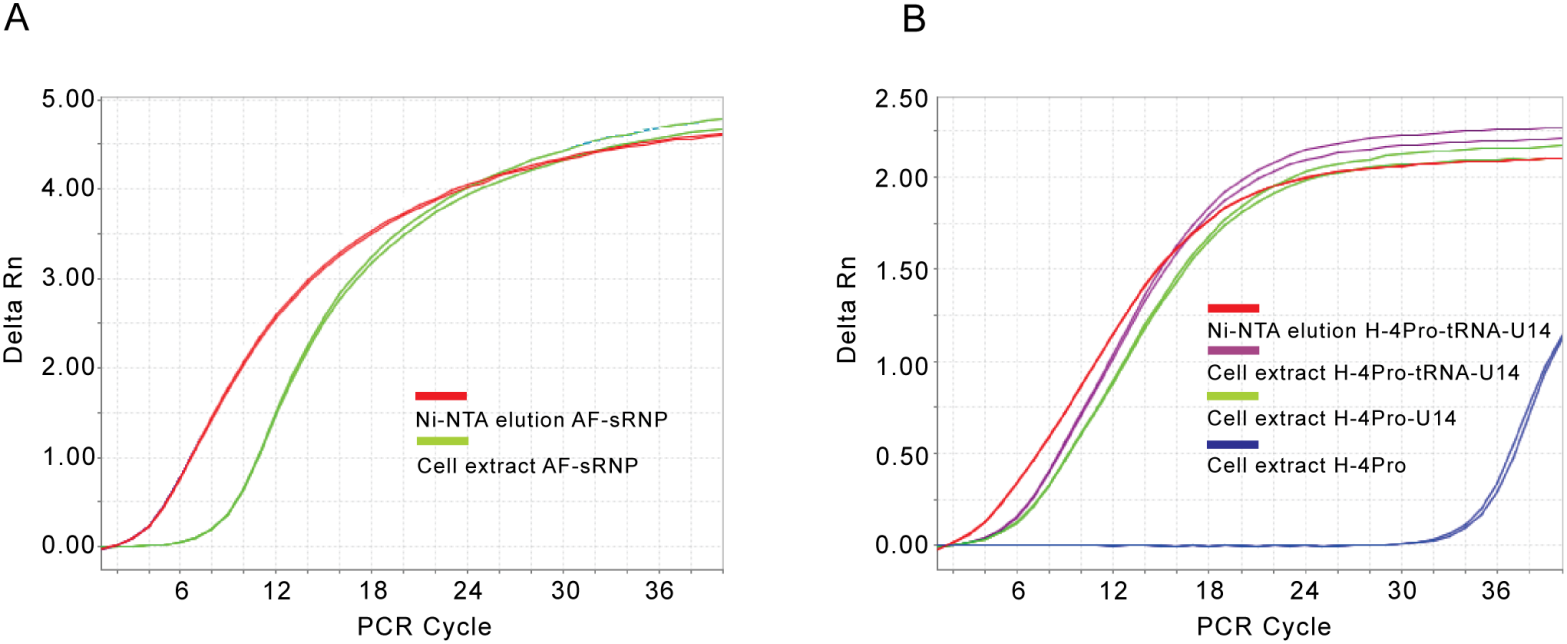
Quantitative RT-PCR analysis of RNA samples from co-expressing box C/D RNPs. Delta Rn indicates the magnitude of amplification and is the normalized and base-line corrected fluorescence emission intensity of the reporter dye obtained for each PCR reaction. A). Amplification plots of qRT-PCR of RNA samples extracted from Ni-NTA purified (red) or cell lysate (green) of *Archaeoglobus fulgidus* sR3 sRNP. B). Amplification plots of qRT-PCR of RNA samples extracted from Ni-NTA purified (red) or cell lysate (green) of human U14 snoRNP.

We were able to purify recombinantly expressed Af box C/D RNP (Figure 3) and human U14 snoRNP (Figure 4) using this strategy. Proteins co-purified with the (tRNA)-sR3 RNA or (tRNA)-U14 snoRNA were verified to be those of the box C/D RNPs based on their positions on SDS-PAGE gel and on mass spectroscopy analysis. More importantly, the RNAs were confirmed by visualization on polyacrylamide gels (Figures 3 & 4) and by qRT-PCR analysis (Figure 5). When total cell lysate were analyzed on polyacrylamide gel stained by SyberGold, RNA molecules corresponding to the sizes of (tRNA)-sR3 and (tRNA)-U14 RNA were present in cells expressing the box C/D proteins and the RNA but were absent in cells expressing only proteins (Figure 3B & 4B). To further verify the presence of co-purified RNA, qRT-PCR was carried out on the RNA extracted from the Ni-NTA elution sample, those from the total cell extract expressing proteins and RNA, and those from the total cell extract expressing proteins only (Figure 5). For the human U14 box C/D snoRNP, qRT-PCR was also carried out on both tRNA-U14 chimera and intact U14 RNA samples. Figure 5A shows specific amplification of sR3 RNA signals using RNA extracted from Ni-NTA elutant and total cell lysate. Figure 5B shows amplification profiles of U14 RNA and a control samples. RNA extracted from Ni-NTA purified human U14 snoRNP and from cells co-expressed snoRNP components with either tRNA-U14 or intact U14 RNA yielded specific signals for U14 RNA. In contrast, qRT-PCR cycles of the total RNA from cells expressing the four proteins only did not yield specific signals for U14 (Figure 5B). The qRT-PCR product from the Ni-NTA elution sample containing tRNA-U14 was further analyzed by TA-cloning and DNA sequencing and found to contain that of U14 RNA (Figure S2).

Comparison of the RNA extracted from total cell lysate between the tRNA-U14- and U14-associated RNPs suggests an important role of the tRNA scaffold in the overall stability of the RNA. The U14 RNA was observed to be at much less quantity as that of the tRNA-U14 under the same expression and purification conditions (Figure 4B). In the case of the sR3 box C/D RNP, the sR3 RNA without tRNA scaffold could not be detected after purification (Figure 3). This is likely due to the heating step in purification that increased the degradation of sR3 RNA.

### Methylation activities of the co-purified box C/D RNPs

**Figure 6:**
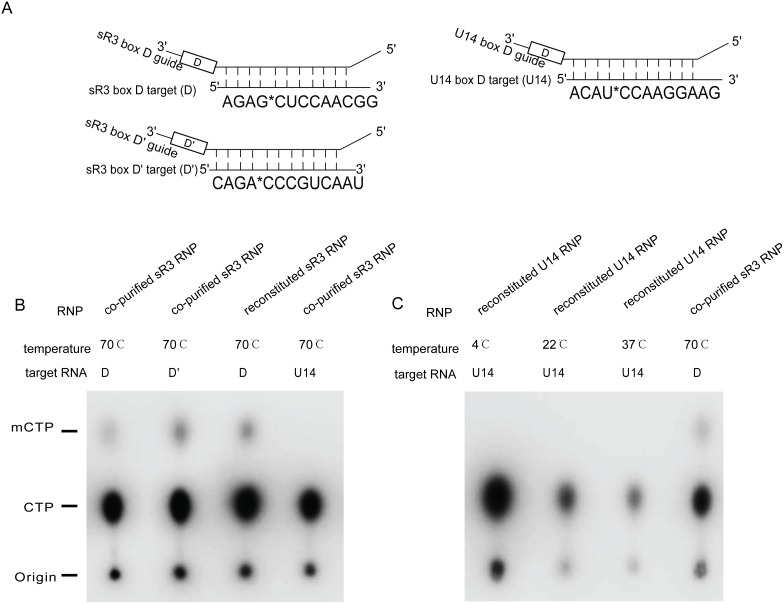
In vitro methylation activity assay results using co-expressed and purified sR3 sRNP and U14 snoRNP. A). Sequences of the target RNAs complementary to upstream sequences of box D of sR3 and U14 and box D′ of sR3 are shown. Radiolabeled nucleotide is indicated by “*C” and is the methylation target nucleotide. B). Various target RNA containing α-^32^P-cytidine were incubated with co-expressed and purified sR3 RNP, or reconstituted RNP control, phenol/chloroform extracted, ethanol precipitated, and RNase P1 digested and separated on a thin layer chromatography plate. The loading position and those of cytidine monophosphate (unmethylated or methylated) were indicated by “Origin”, “CMP” and “mCMP”, respectively. The target RNA complementary to box D, D′ guide of sR3 RNA, and to box D guide of U14 RNA are indicated by “D”, “D′”, “U14”, respectively. C). Methylation activity analysis of U14 snoRNP at different temperatures (Lanes 1–3). Co-purified sR3 sRNP with target RNA complementary to its box D is included as a positive control (Lane 4).

The assay of 2′-O-methyltransferase activity showed that the co-purified Af sR3 sRNP by our pQlink-N-based plasmid system is fully functional. The target RNA complementary to either D or D′ guide was observed to acquire a methyl group after incubating with the purified sR3 sRNP (Figure 6A & 6B). In contrast, a substrate RNA complementary to U14 snoRNA was not methylated under the same reaction condition (Figure 6B). However, we did not detect any methylation activity under multiple conditions on the target RNA complementary to U14 RNA using the co-purified human U14 snoRNP (Figure 6C).

## Discussion

In this study, we combined the versatile pQLink system and the tRNA fusion method to co-express the archaeal and the human box C/D RNP complex in *E. coli*. This strategy provides several advantages over the method of preparing RNPs from individually expressed components. The first is the increased stability of the otherwise unstable protein components. Individually expressed human NOP56, NOP58 and fibrillarin are not suitable for purification due to insolubility [21]. In contrast, co-expressed human box C/D proteins were stabilized to allow their purification. The second advantage of this system is the increased stability of the RNA. Without the tRNA scaffold, U14 RNA could not be co-purified with the proteins. Thirdly, the pQLink system provides a versatile platform for purification and expansion of large RNP complexes. The co-expression system introduces a single affinity tag that allows a single step purification of the intact complex. Finally, the pQLink vector can in theory accommodate unrestricted number of proteins/RNAs, unlike other systems such as poly-cistronic constructs that are limited by a single promoter [35] and the pETDuet vectors that are limited to two coding sequences [36].

In eukaryotes, box C/D snoRNPs are maturated and assembled through a highly complex process and require multiple biogenesis factors. Examples of the biogenesis factors that directly interact with box C/D snoRNP include a co-chaperone of Hsp90, the R2TP complex, that comprises of Tah1, Pih1, and two AAA+ ATPases Rvb1 and Rvb2 [22], [23], [37]–[39], and NUFIP (Rsa1p in yeast) [20], [21], [40], Other factors such as TGS1, exosome, La, and Lsm proteins are known to assist processing and CBC, PHAX, CRM1, Ran and NOPP140 are involved in nucleocytoplasmic transport of at least one snoRNP [21], [22], [41]–[43]. In consideration of the fact that our cloning constructs allow incorporation of multiple coding sequences, they provide a possible means to study the function of these snoRNP assembly factors by creating a variety of co-expression combinations. These constructs may be used to identify pair-wise interactions or roles of the assembly factors in the production of functional snoRNP particles.

In order to assess the quality of co-purified snoRNPs, we analyzed the methylation activity of box C/D complexes by an in vitro RNA-guided 2′-O-methylaton assay. We found that while the co-purified Af sR3 sRNP was fully active, the co-purified human U14 box C/D snoRNP did not show any detectable activity. This result suggests a requirement for additional factors in U14 box C/D snoRNP assembly that are lacking in the current expression system. Consistently, while 15.5 K protein has significant expression, it is only partially co-purified with the complex (Figure 4A), indicating its low level of association with the snoRNP. Previous studies show that Rvb1, Rvb2 and NUFIP (Rsa1p) can bridge interactions between 15.5 K/Snu13p and the other core proteins [22], [40]. Moreover, Rvb1 and Rvb2 are believed to participate in the structural rearrangement of the box C/D snoRNP at the expense of ATP binding or hydrolysis [22]. Thus, the recombinantly co-expressed and co-purified U14 snoRNP is still not assembled correctly and factors such as Rvb1, Rvb2 and NUFIP are likely required for its correct assembly. Having a versatile co-expression system available, it is possible to assess the functional roles of the assembly factors in U14 snoRNP assembly.

## Supporting Information

Figure S1 Sequence alignment of human NOP56 (human_N56), human NOP58 (human_N58) against their homologs: *Saccharomyces cerevisiae* Nop56p (Sacc_N56), *Saccharomyces cerevisiae* Nop58p (Sacc_N58), *Pyrococcus furiosus* Nop56/58 (Pyrococcus), and *Sulfolobus solfataricus* Nop56/58 (Sulfolobus). Red boxes highlight residues that have strict sequence identity and blue boxes indicate residues that have sequence similarity. Secondary structure elements of *Pyrococcus furiosus* Nop56/58 are derived from its crystal structure (PDBid: 3NMU).
(DOCX)

Figure S2 Validation of human U14 RNA by cloning the reverse PCR product obtained from co-purified human box C/D RNP followed by DNA sequencing. Human U14 snoRNA sequence cloned in vector pCR2.1 is highlighted in yellow.
(DOCX)
