# Supplementary figures and images for: Co-Expression and Co-Purification of Archaeal and Eukaryal Box C/D RNPs
Source: PLoS One. 2014 Jul 31;9(7):e103096. doi: 10.1371/journal.pone.0103096 (PMC4117494; doi:10.1371/journal.pone.0103096)

**Figure S1 Alignment of NOP56/58 homologs**.


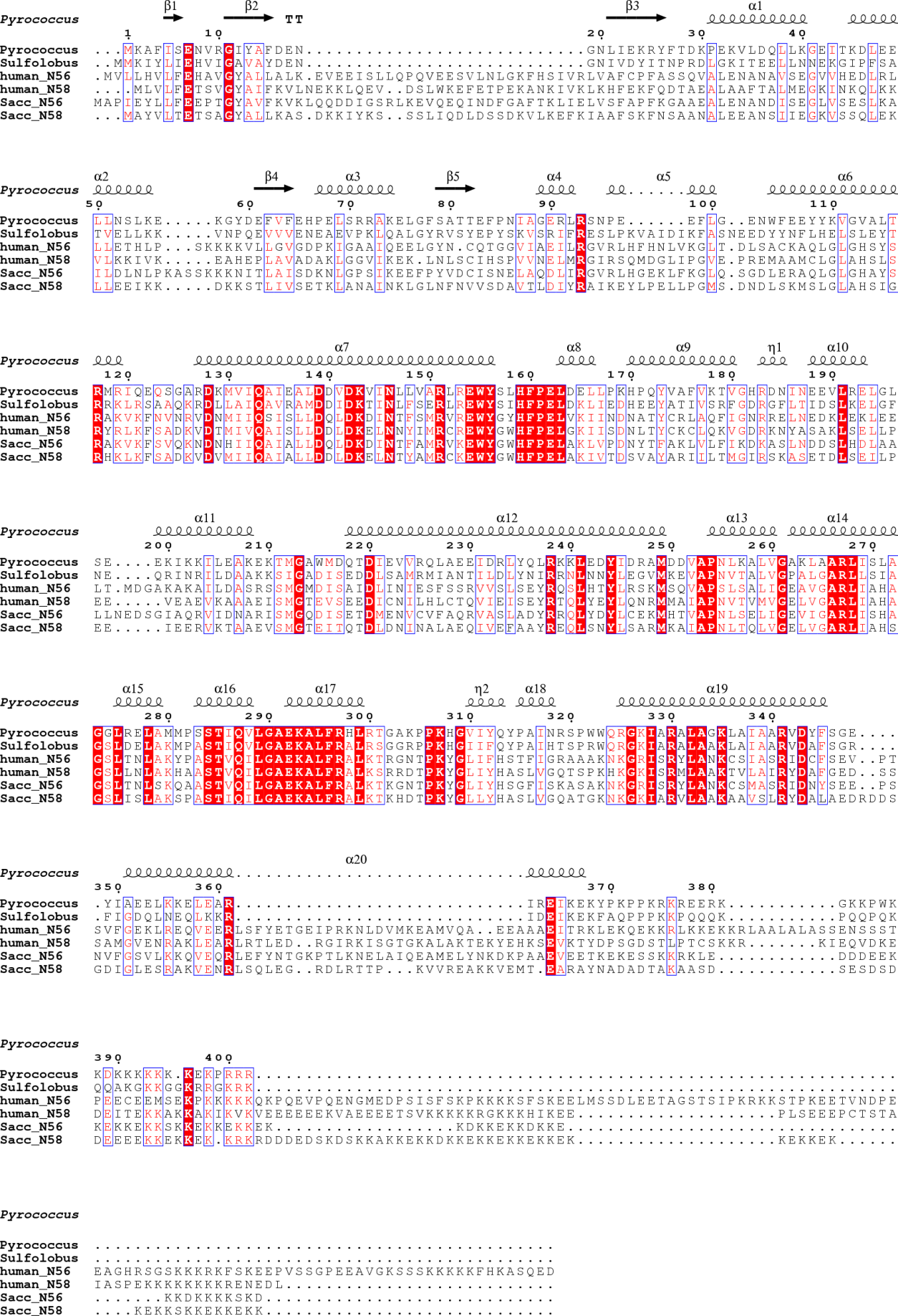

Supplement: Figure S1 — Sequence alignment of human NOP56 (human_N56), human NOP58 (human_N58) against their homologs: Saccharomyces cerevisiae Nop56p (Sacc_N56), Saccharomyces cerevisiae Nop58p (Sacc_N58), Pyrococcus furiosus Nop56/58 (Pyrococcus), and Sulfolobus solfataricus Nop56/58 (Sulfolobus). Red boxes highlight residues that have strict sequence identity and blue boxes indicate residues that have sequence similarity. Secondary structure elements of Pyrococcus furiosus Nop56/58 are derived from its crystal structure (PDBid: 3NMU). (DOCX) [file pone.0103096.s001.docx]
