# Supplementary material for: Co-Expression and Co-Purification of Archaeal and Eukaryal Box C/D RNPs
Source: PLoS One. 2014 Jul 31;9(7):e103096. doi: 10.1371/journal.pone.0103096 (PMC4117494; doi:10.1371/journal.pone.0103096)
Supplement: Figure S2 — Validation of human U14 RNA by cloning the reverse PCR product obtained from co-purified human box C/D RNP followed by DNA sequencing. Human U14 snoRNA sequence cloned in vector pCR2.1 is highlighted in yellow. (DOCX) [file pone.0103096.s002.docx]

**Figure S2 TA-Cloning Sequence of human U14 snoRNA**

ATTTCTCTCTCTTAAGACGCGGTAGAGGTTTCGTTGCAAATGAAGGCGTCAGGACCGTGGAAAGGATTCAAGGATCTCTGGATCCTTTTCTGCGGCGTATCGCTGCTGCAACAAAACAACCGCTCTACCAGCGTGTTGTGGCGATCAGAGCTACACTCTTTTCCGAGTACTGCTCAGCAGAGCGCAGATACCAATACTGTCTTCTAGTGTAGCCGTAGTAGGCCACACTCAAGAACTCTGTAGCACGCTACATACTCGCTCTGCTAATCCTGTACAGTGGCTGCTGCCAGTGGCGATAAGTCGTGTCTTACCGGGTTGGACTCAAGACGATAGTTACCGGATAAGGCGCAGCGGTCGGGCTGAACGGGGGGTTCGTGCACACAGCCCAGCTTGGAGCGAACGACCTACACCGAACTGAGATACCTACAGCGTGAGCTATGAGAAAGCGCCACGCTTCCCGAAGGGAGAAAGGCGGACAGGTATCCGGTAAGCGGCAGGGTCGGAACAGGAGAGCGCACGAGGGAGCTTCCAGGGGGAAACGCCTGGTATCTTTATAGTCCTGTCGGGTTTCGCCACCTCTGACTTGAGCGTCGATTTTTGTGATGCTCGTCAGGGGGGCGGAGCCTATGGAAAAACGCCAGCAACGCGGCCTTTTTACGGTTCCTGGCCTTTTGCTGGCCTTTTGCTCACATGTTCTTTCCTGCGTTATCCCCTGATTCTGTGGATAACCGTATTACCGCCTTTGAGTGAGCTGATACCGCTCGCCGCAGCCGAACGACCGAGCGCAGCGAGTCAGTGAGCGAGGAAGCGGAAGAGCGCCCAATACGCAAACCGCCTCTCCCCGCGCGTTGGCCGATTCATTAATGCAGCTGGCACGACAGGTTTCCCGACTGGAAAGCGGGCAGTGAGCGCAACGCAATTAATGTGAGTTAGCTCACTCATTAGGCACCCCAGGCTTTACACTTTATGCTTCCGGCTCGTATGTTGTGTGGAATTGTGAGCGGATAACAATTTCACACAGGAAACAGCTATGACCATGATTACGCCAAGCTTGGTACCGAGCTCGGATCCACTAGTAACGGCCGCCAGTGTGCTGGAATTCGCCCTTCACTGTGATGATGGTTTTCCAACATTCGCAGTTTCCACCAGAAAGGTTTTCCTTAGTGTTGGGTAAACCTTCCTTGGATGTCTGAGTGAAAGGGCGAATTCTGCAGATATCCATCACACTGGCGGCCGCTCGAGCATGCATCTAGAGGGCCCAATTCGCCCTATAGTAGTCGTTACGC
